# Cyclopeptide moroidin inhibits vasculogenic mimicry formed by glioblastoma cells *via* regulating β-catenin activation and EMT pathways

**DOI:** 10.7555/JBR.38.20240015

**Published:** 2024-05-29

**Authors:** Pengxiang Min, Yingying Li, Cuirong Wang, Junting Fan, Shangming Liu, Xiang Chen, Yamin Tang, Feng Han, Aixia Zhang, Lili Feng

**Affiliations:** 1 Key Laboratory of Cardiovascular & Cerebrovascular Medicine, International Joint Laboratory for Drug Target of Critical Illnesses, School of Pharmacy, Nanjing Medical University, Nanjing, Jiangsu 211166, China; 2 Department of Pharmaceutical Analysis, School of Pharmacy, Nanjing Medical University, Nanjing, Jiangsu 210029, China; 3 Department of Analysis and Testing Center, School of Basic Medical Sciences, Nanjing Medical University, Nanjing, Jiangsu 211166, China; 4 Institute of Brain Science, the Affiliated Brain Hospital of Nanjing Medical University, Nanjing, Jiangsu 211166, China; 5 Department of Clinical Pharmacology, School of Pharmacy, Nanjing Medical University, Nanjing, Jiangsu 211166, China

**Keywords:** moroidin, vasculogenic mimicry, glioblastoma, EMT, β-catenin

## Abstract

Glioblastoma (GBM) is a highly vascularized malignant brain tumor with poor clinical outcomes. Vasculogenic mimicry (VM) formed by aggressive GBM cells is an alternative approach for tumor blood supply and contributes to the failure of anti-angiogenic therapy. To date, there is still a lack of effective drugs that target VM formation in GBM. In the present study, we evaluated the effects of the plant cyclopeptide moroidin on VM formed by GBM cells and investigated its underlying molecular mechanisms. Moroidin significantly suppressed cell migration, tube formation, and the expression levels of α-smooth muscle actin and matrix metalloproteinase-9 in human GBM cell lines at sublethal concentrations. The RNA sequencing data suggested the involvement of the epithelial-mesenchymal transition (EMT) pathway in the mechanism of moroidin. Exposure to moroidin led to a concentration-dependent decrease in the expression levels of the EMT markers N-cadherin and vimentin in GBM cells. Moreover, moroidin significantly reduced the level of phosphorylated extracellular signal-regulated protein kinase (p-ERK) and inhibited the activation of β-catenin. Finally, we demonstrated that the plant cyclopeptide moroidin inhibited VM formation by GBM cells through inhibiting the ERK/β-catenin-mediated EMT. Therefore, our study indicates a potential application of moroidin as an anti-VM agent in the treatment of GBM.

## Introduction

Glioblastoma (GBM) is the most aggressive and lethal primary brain tumor in adults, with a median survival of less than two years^[1]^. Because hypervascularization is a hallmark of GBM and is one of the main causes of poor prognosis, anti-angiogenic therapies (AAT) have been used as adjuvants to current standard therapies^[2–3]^. However, some emerging evidence indicates that bevacizumab (BEV) fails to improve the overall survival of patients because of drug resistance or rapid relapse^[4]^. The poor efficacy of AAT is largely attributed to the complexity of tumor vascular networks.

It is now well-established that tumor vasculogenesis is not exclusively attributable to endothelial cells. The tumor cells themselves mimic endothelial function by acquiring plasticity, forming vasculogenic-like networks that provide blood supply for tumor growth and metastasis^[5–7]^. This endothelial cell-independent microcirculation pattern is named vascular mimicry (VM). Studies reported VM in gliomas for the first time in 2005^[8]^. Further investigations have validated that VM is significantly correlated with high grades of tumor malignancy and invasiveness, and is associated with a poor clinical prognosis in GBM^[9–10]^. The reason for the poor efficacy and resistance of classical antiangiogenic drugs may be because these drugs are ineffective against VM, and even induce VM channels in GBM^[11]^. In light of the evidence, it is imperative to develop novel therapeutics that target VM to overcome AAT resistance and control the recurrence.

There has been an increasing evidence that phytochemicals, such as curcumin, berberine, and daurisoline, inhibit cell migration, invasion, and VM formation by reducing matrix metalloproteinases (MMPs) expression levels or inhibiting epithelial-mesenchymal transition (EMT) of tumor cells^[12–13]^. Cyclopeptides have attracted the attention of both pharmacists and investigators because of their distinct therapeutic and pharmacokinetic properties that differ from those of small compounds and antibodies. Moroidin is a natural heterodicyclopeptide isolated from the seeds of *Celosia argentea*, with the activity to inhibit microtubule polymerization, which has been considered a novel microtubule targeting agent^[14–15]^. In a previous study, we demonstrated that moroidin induced the G2/M phase arrest and apoptosis of A549 cancer cells. Moroidin also significantly reduced the migration and invasion of A549 cells at sublethal concentrations^[16]^. However, the activity of moroidin against GBM has barely been investigated.

In the present study, we aimed to investigate the effects of moroidin on VM formed by GBM, to study its underlying molecular mechanisms, and to provide a potential drug candidate for GBM treatment and reference information for AAT. Such a study may offer new approaches for addressing resistance to the reported angiogenesis inhibitors^[17]^.

## Materials and methods

### Chemicals and reagents

Moroidin was isolated from *Celosia* *argentea* and characterized by Dr. Jun-ting Fan at Nanjing Medical University^[16]^ and then dissolved to 20 mmol/L in dimethyl sulfoxide (DMSO). GAPDH (1∶10000; Cat. #2118), N-cadherin (1∶1000; Cat. #13116), β-catenin (1∶1000; Cat. #8480), vimentin (1∶1000; Cat. #5741), SNAIL (1∶1000; Cat. #3879), extracellular signal-regulated protein kinase (ERK; 1∶1000; Cat. #4695), p-ERK (1∶1000; Cat. #4370) antibodies were purchased from Cell Signaling Technology (Danvers, MA, USA). α-SMA (1∶1000; Cat. #sc-53142), E-cadherin (1∶1000; Cat. #sc-21791), and TWIST (1∶1000; Cat. #sc81417) antibodies were purchased from Santa Cruz Biotechnology (Dallas, CA, USA). Anti-active β-catenin (1∶1000; Cat. #05-665) was purchased from Millipore (Billerica, MA, USA). Anti-MMP-9 (1∶1000; Cat. #27306-1-AP) was purchased from Proteintech (Wuhan, China).

### Cell culture

Human GBM cell line U87 and human umbilical vein endothelial cell line HUVEC were purchased from the American Type Culture Collection (ATCC, Rockville, MD, USA). Human GBM cell line U251 was purchased from Procell Life Science Technology Co., Ltd. (Wuhan, China). Cells were cultured in DMEM (high glucose; Gibco, Carlsbad, CA, USA) with 10% fetal bovine serum (Gibco), penicillin (100 U/mL) and streptomycin (100 μg/mL) under standard culture conditions (humidified air containing 5% CO_2_ at 37 ℃).

### Cell Counting Kit-8 (CCK8) assay

Cells (3000/well) were seeded in 96-well plates and incubated with various concentrations of moroidin for 24 h or 48 h. Then the cell viability was determined using Cell Counting Kit-8 (CCK-8, Biosharp, Hefei, China) according to the manufacturer's instructions. Briefly, 10 µL of CCK-8 solution was added to each well following an incubation at 37 ℃ for 1 h. Subsequently, the absorbance at 450 nm was measured using a microplate reader.

### Cell scratch assay

The cell scratch assay was performed as previously reported with slight modifications^[18]^. Briefly, when the cell confluency reached 80%, the monolayers were scratched and then cultured with various doses of moroidin (0, 0.5, 1, or 2 μmol/L) for 24 h. The wounded area was imaged at 0 and 24 h by using a microscope (Leica Microsystems, Wetzlar, Germany) and quantified by measuring the area with the ImageJ software.

### Transwell assay

Cell migration was measured by using Transwell Chambers with 8-μm pores (Corning, NY, USA). Cells were treated with 0, 0.5, 1, or 2 μmol/L of moroidin for 24 h and then seeded into the upper chamber for another 24 h. The cells were fixed with 4% paraformaldehyde (PFA) and stained with 0.2% crystal violet. Nonmigrating cells in the upper chamber were wiped with cotton swabs. The number of stained cells on the underside of the membrane was counted in photos with the Cell Counter plug-in of the ImageJ software.

### Tube formation assay

Matrigel (growth factor reduced; Corning) was coated on 96-well plates (150 μL/well) and incubated at 37 ℃ for 1 h. Cells were treated with various doses of moroidin (0, 0.5, 1, or 2 μmol/L) for 24 h and then seeded onto the surface of Matrigel. After incubation for 8 h, the structures formed by GBM cells were observed under a microscope. The tubules were quantified by measuring the number of meshes, master segments, and master junctions by using the Angiogenesis Analyze plug-in of the ImageJ software.

### Western blotting

After determining the protein concentration with a Lowry Protein Assay Kit (Bio-Rad, Hercules, CA, USA), the collected cell lysates were separated by using 10% SDS-PAGE and transferred onto PVDF membranes (Millipore). Subsequently, the membranes were blocked with 5% non-fat milk and immunodetected with primary antibodies. After overnight incubation at 4 ℃, the membranes were incubated with HRP-conjugated secondary antibody and detected with an ECL kit (Biological Industries, Israel). Densitometry analysis was performed by using the ImageJ software, and band intensities were normalized to GAPDH.

### Real-time reverse transcription PCR (qRT-PCR)

Total RNA was extracted using the TRIzol reagent (Takara, Kyoto, Japan) according to the manufacturer's protocol. The corresponding cDNA was synthesized using a cDNA Synthesis SuperMix for qPCR Kit (Vazyme, Nanjing, China). qPCR was performed on an ABI 7500 Fast Real-Time PCR System (Applied Biosystems, Foster City, CA) using SYBR Green Master Mix (Vazyme). The PCR primers are listed in ***Table 1***. RNA expression levels were evaluated using the 2^−ΔΔCt^ method by normalizing to *GAPDH*.

**Table 1:** The primer sequences for real-time reverse transcription PCR

Genes	Orientations	Sequences (5′-3′)
*MMP2*	Forward Reverse	GTGAAGTATGGGAACGCCG GCCGTACTTGCCATCCTTCT
*MMP9*	Forward Reverse	TACTGTGCCTTTGAGTCCG TTGTCGGCGATAAGGAAG
*MMP14*	Forward Reverse	GCAGAAGTTTTACGGCTTGCAA CCTTCGAACATTGGCCTTGAT
*GAPDH*	Forward Reverse	CATCAGCAATGCCTCCTGCAC TGAGTCCTTCCACGATACCAAAGTT

### Immunofluorescence staining

Cells were fixed with 4% PFA for 15 min, incubated with 0.1% Triton X-100 for 10 min, and blocked with 5% TSA for 1 h. After binding with β-catenin antibody, followed by a second antibody, and stained with DAPI, cells were photographed using a confocal microscope (Carl Zeiss Meditec, Jena, Germany).

### RNA-sequence and data analysis

Total RNA was extracted using RNAiso Plus (Takara, Kyoto, Japan), followed by quality control using an Agilent 2100 Bioanalyzer (Agilent Technologies, Santa Clara, CA). An AMPure XP system (Beckman Coulter, Beverly, MA, USA) was used to select qualified samples for next-generation sequencing. The library was sequenced on a NovaSeq 6000 platform (Illumina) by Shanghai Personal Biotechnology Co., Ltd. The fragments per kilobase of exon model per million mapped fragments (FPKM) was used to standardize differences of genes and was analyzed using DESeq (version 1.30.0) with the following screen inclusion conditions: expression difference multiple |log_2_(fold change)| > 1, significant *P*-value < 0.05.

Gene set enrichment analysis (GSEA, version 4.1.0) was performed using the MSigDB gene sets (h.all.v7.2.symbols.gmt) and (c2.cp.kegg.v7.4.symbols.gtm) gene sets for indicating the Hallmarks and Kyoto Encyclopedia of Genes and Genomes (KEGG) pathways. Normalized Enrichment Score (NES) was calculated as the primary statistic for GSEA and rank genes by using a measure of absolute "signal-to-noise" value.

By using topGO to perform the gene ontology (GO) enrichment analysis on differential genes, we calculated the *P*-value with the hypergeometric distribution method (the standard of significant enrichment is *P*-value < 0.05). The GO term with significantly enriched differential genes was used to determine the main biological functions performed by differential genes.

### Statistical analysis

All experiments were performed at least three times. Data were analyzed using GraphPad Prism software and presented as mean ± standard deviation. Statistical comparisons were analyzed using one-way ANOVA followed by Dunnett's post hoc test or two-way ANOVA followed by Dunnett's post hoc test. *P* < 0.05 was considered statistically significant.

## Results

### Cytotoxic effects of moroidin on GBM cells

**Figure 1:**
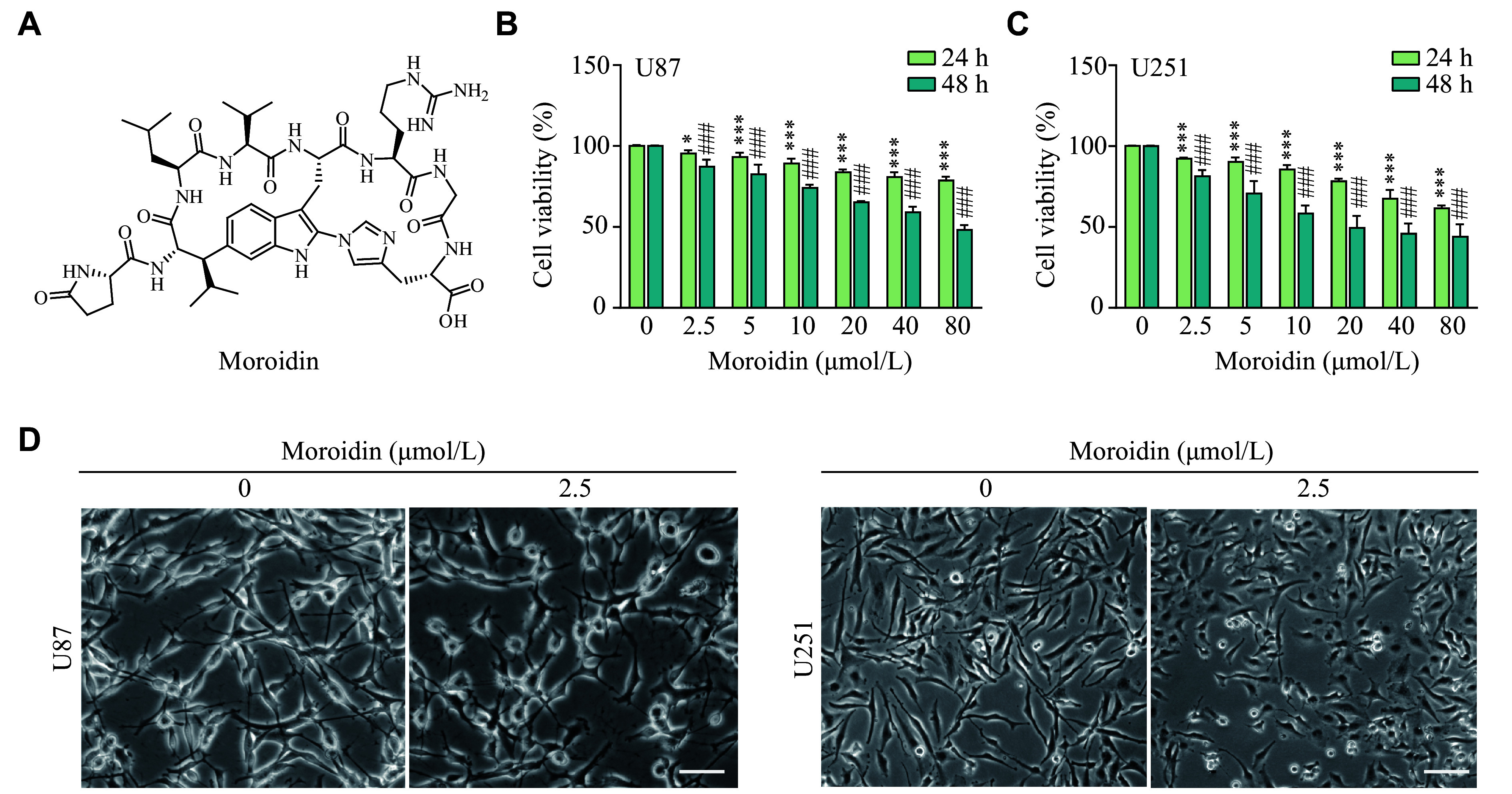
Cytotoxic effects of moroidin on glioblastoma cells.

To evaluate the activity of moroidin (***Fig. 1A***) on GBM, U87, and U251 cells were treated with moroidin for 24 and 48 h, and survival rates were determined using CCK8 assays. As shown in ***Fig. 1B*** and ***Fig. 1C***, moroidin slightly decreased the viability of U87 and U251 cells at low concentrations at both 24 and 48 h after treatment. The survival rate of both cell lines was over 90% when incubated with moroidin at concentrations of 2.5–5 μmol/L for 24 h. However, the morphology of U87 and U251 cells changed from a long and spindle shape to a polygonal shape induced by 2.5 μmol/L moroidin treatment for 24 h (***Fig. 1D***), indicating the distinct effect of moroidin on GBM cells at relatively low concentrations rather than cytotoxicity. In addition to GBM cells, moroidin also displayed a low toxicity on the endothelial cell line HUVEC at low concentrations (***Supplementary Fig. 1***, available online). These results indicate the safety of moroidin as a potential VM modulating agent.

### Moroidin inhibited the motility of GBM cells

**Figure 2:**
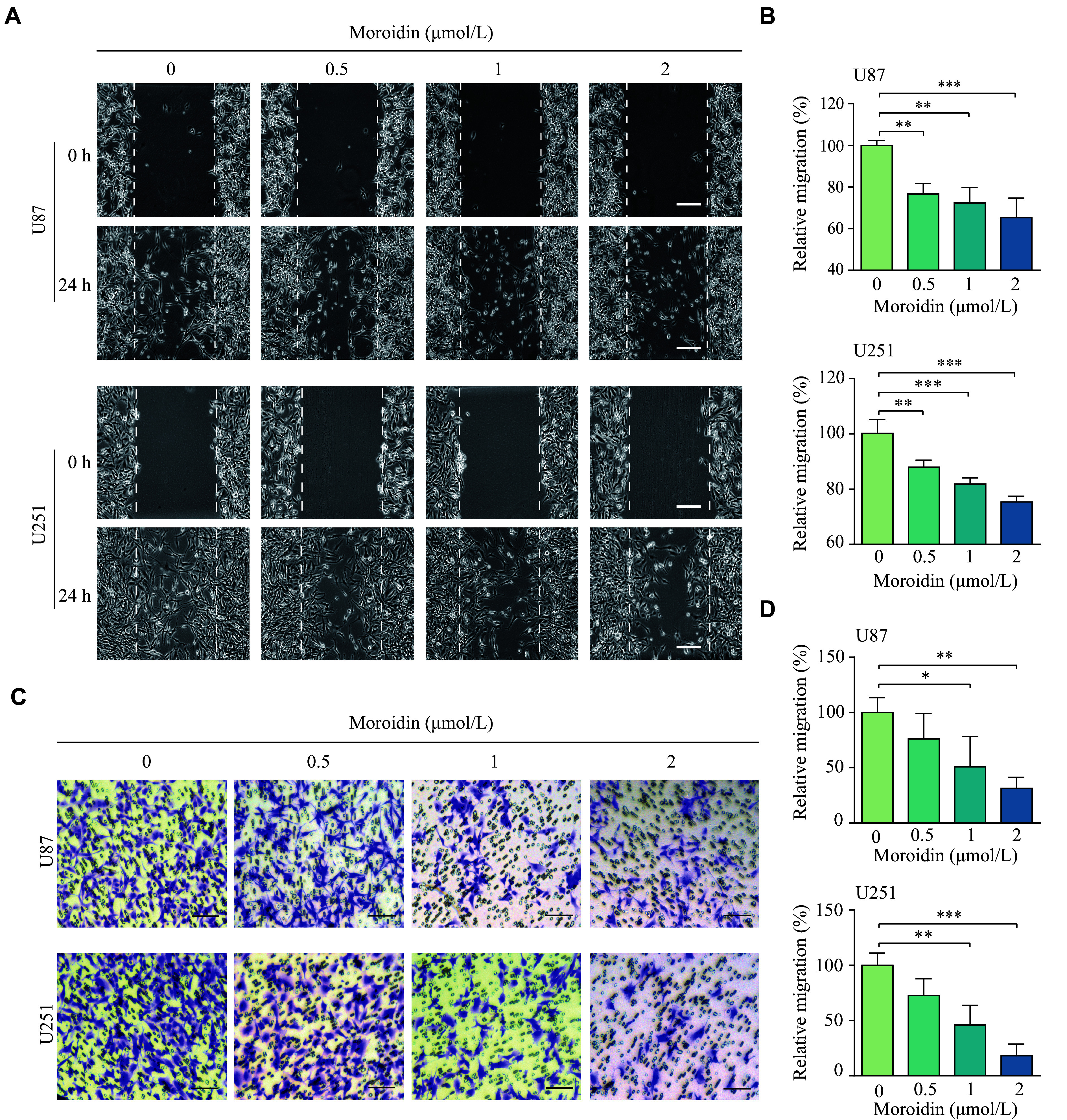
Moroidin inhibited glioblastoma cell motility.

The effect of moroidin on cell motility was further investigated using wound scratch and Transwell assays. As shown in ***Fig. 2A*** and ***2B***, treatment with lower doses of moroidin (0.5, 1, and 2 μmol/L) resulted in a significant decrease in the migration distances of both U87 and U251 cells. The number of migrated GBM cells was also reduced by moroidin treatment in a dose-dependent manner (***Fig. 2C*** and ***2D***). These data indicate the inhibition of moroidin on cell migration at sub-lethal concentrations.

### Moroidin suppressed the VM formation

**Figure 3:**
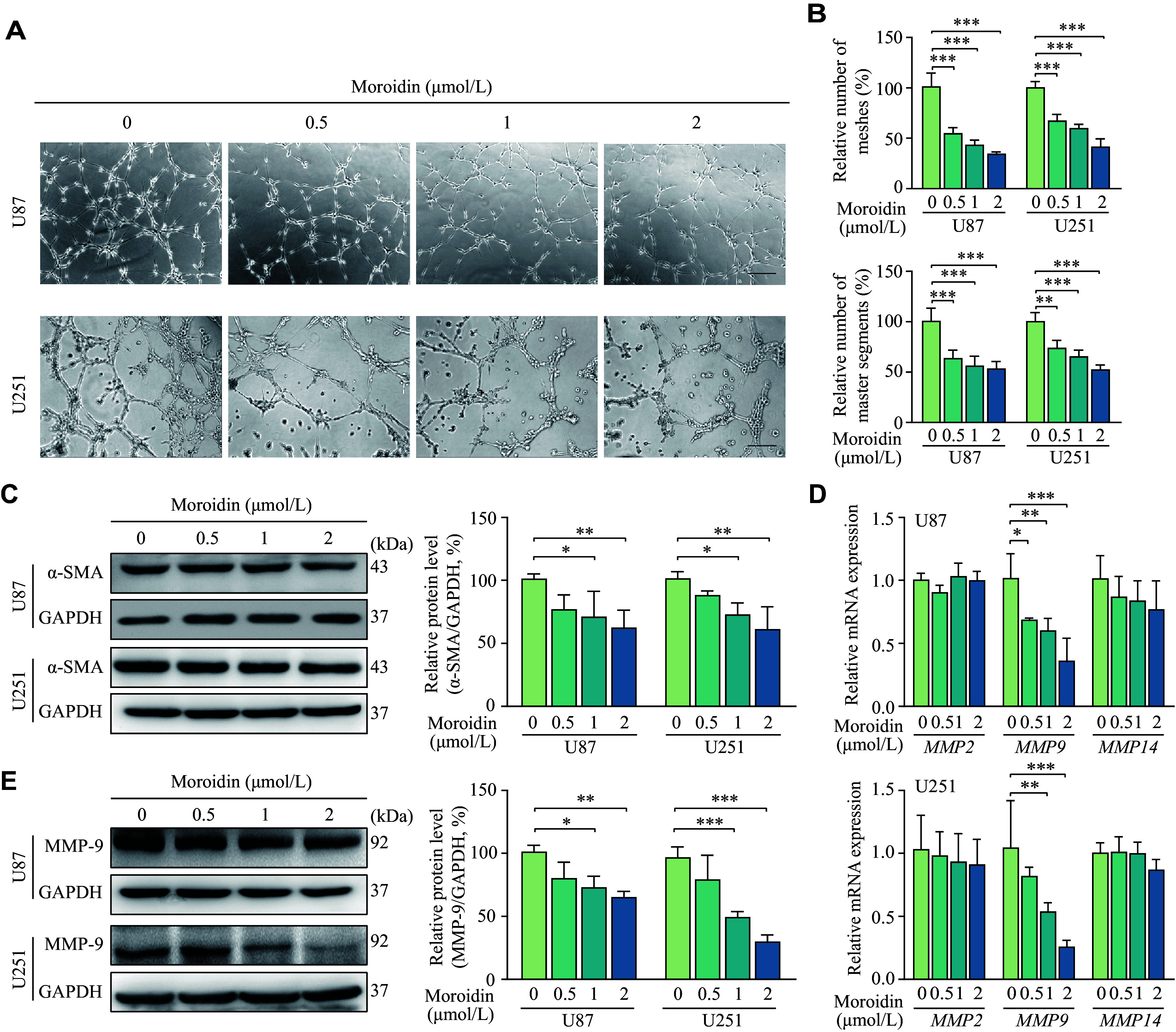
Effects of moroidin on vasculogenic mimicry formation.

To determine the effect of moroidin on VM formation directly, an *in vitro* Matrigel tube formation assay was performed. U87 and U251 cells efficiently formed vasculogenic-like networks on Matrigel, which were significantly inhibited by moroidin at lower doses. Compared with the U87 cells, the numbers of meshes were reduced to 55.26%, 43.82%, and 34.51% in the 0.5, 1, and 2 μmol/L moroidin-treated groups, respectively, while master segments were reduced to 64.49%, 57.07%, and 54.01%, respectively (***Fig. 3A*** and ***3B***). Similar results were observed in the U251 cells treated with the same concentrations and incubation time of moroidin (***Fig. 3A*** and ***3B***). However, BEV was found to enhance the tube formation, which was further disrupted in the presence of moroidin (***Supplementary Fig. 2***, available online).

Expression levels of the VM marker α-SMA were significantly decreased after moroidin administration in both U87 and U251 cells (***Fig. 3C***). Considering the close association of MMP-2, MMP-9, and MMP-14 with the GBM VM formation, we then examined the effects of moroidin on the mRNA levels of these MMPs. The results showed that moroidin had no effect on the expression levels of *MMP2* and *MMP14*, but significantly downregulated the expression levels of *MMP9* (***Fig. 3D***). The results of Western blotting also verified that moroidin reduced the protein levels of MMP-9 in both U87 and U251 cells (***Fig. 3E***). These data suggest that moroidin inhibited VM in GBM cells.

### Moroidin repressed EMT in GBM cells

**Figure 4:**
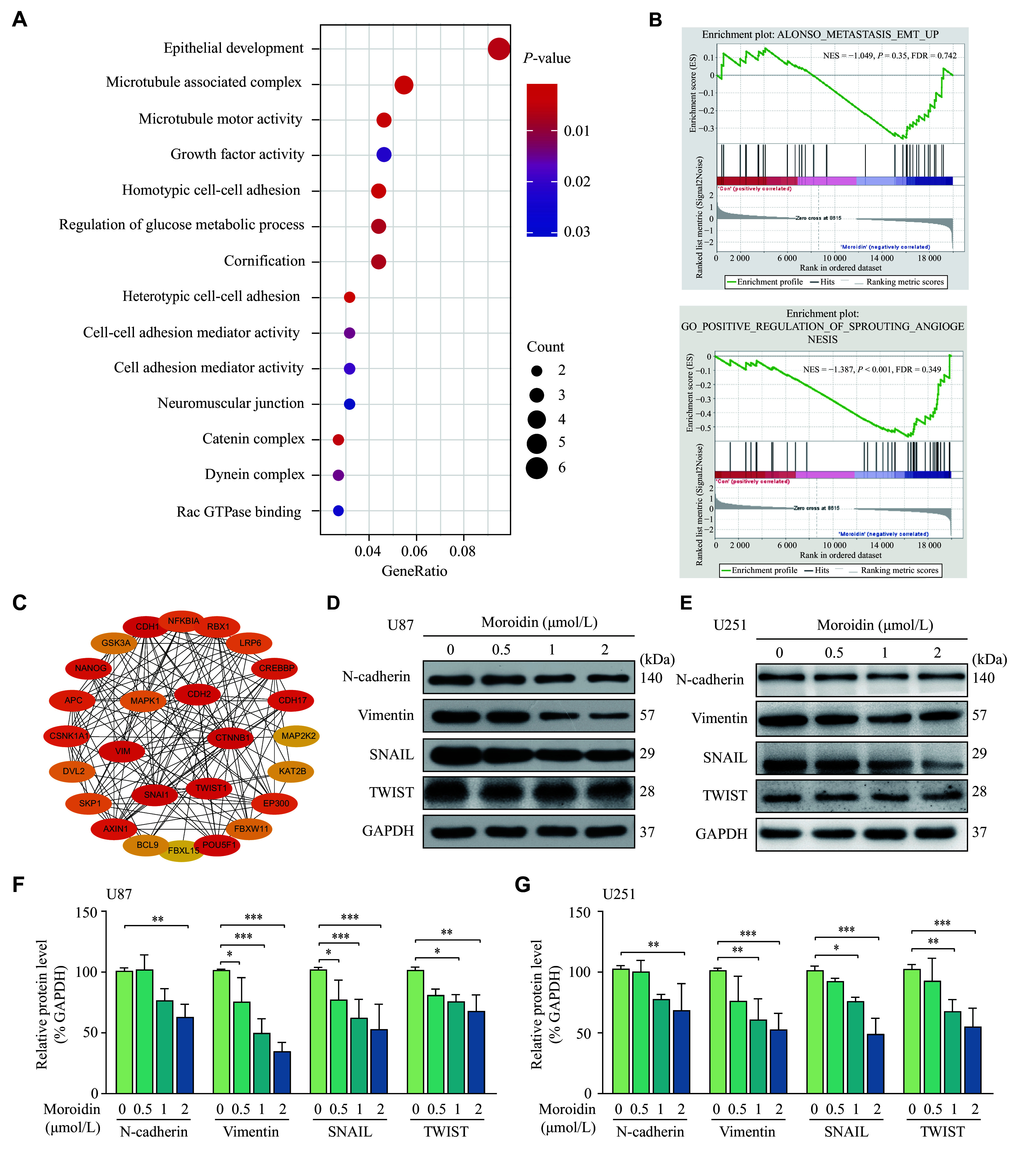
Moroidin down-regulated the expression levels of epithelial-mesenchymal transition (EMT)-related proteins.

U87 cells were treated with 2 μmol/L moroidin or the same volume of DMSO for 24 h, and then RNA-sequencing was performed to comprehensively determine the molecular mechanism of moroidin on GBM cells. The GO analysis data showed that the enriched genes were mainly involved in both epithelial cell development and the microtubule-related complexes (***Fig. 4A***). GSEA revealed that genes involved in the EMT-related tumor metastasis and angiogenesis were downregulated in moroidin-treated U87 cells, compared with those in control cells (***Fig. 4B***). The potential interactions between EMT-related proteins were also predicted using the online database STRING (http://string-db.org; ***Fig. 4C***). These results indicate new functions of moroidin other than as a microtubule-targeting agent.

Further experiments were performed to study the effect of moroidin on the EMT process in U87 and U251 cells. The Western blotting results showed that moroidin dose-dependently decreased the expression levels of mesenchymal cell markers N-cadherin and vimentin, as well as the expression levels of SNAIL and TWIST, the key EMT transcription factors (***Fig. 4D***–***4G***). Additionally, the protein level of E-cadherin was elevated when incubated with moroidin (***Supplementary Fig. 3***, available online). These data suggest that moroidin inhibited VM in GBM cells by inhibiting the EMT pathway.

### Moroidin restrained the EKR/β-catenin signal

**Figure 5:**
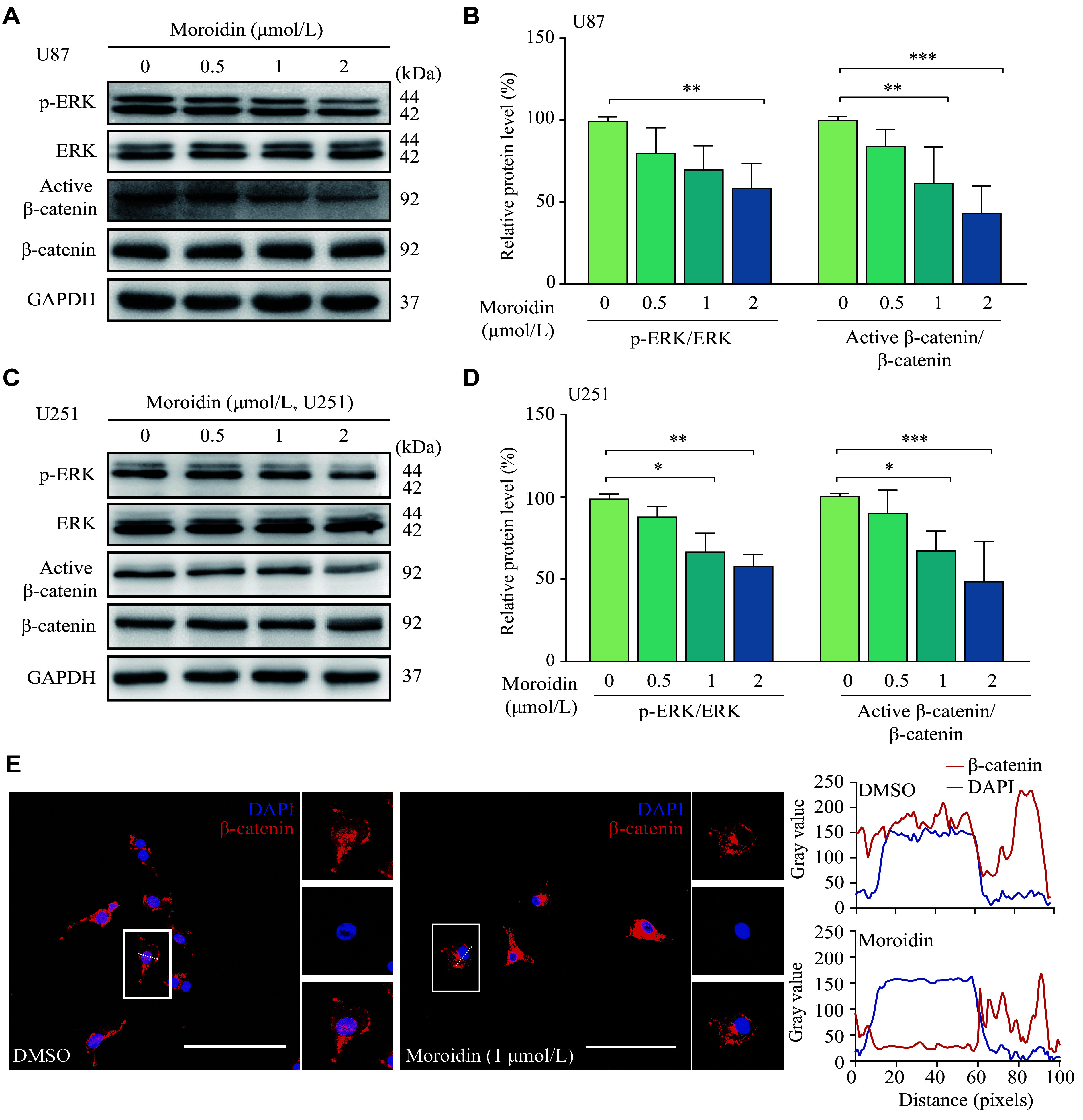
Moroidin repressed the activation of the ERK/β-catenin signaling pathway.

To elucidate the molecular mechanism through which moroidin inhibited EMT in GBM cells, we investigated the upstream key regulators of EMT. It was found that the expression levels of active β-catenin were significantly decreased after the administration of moroidin in a dose-dependent manner (***Fig. 5A***–***5D***). The data from immunofluorescence assay revealed that β-catenin was distributed in the cell membrane, cytoplasm, and nucleus of the control cells. However, in the moroidin-treated cells, β-catenin mainly accumulated in the cytoplasm, and its nuclear distribution was significantly lower than that in the control cells (***Fig. 5E*** and ***Supplementary Fig. 4*** [available online]). These results suggest that moroidin inhibited the activation of β-catenin.

ERK has been reported to be an important regulator for both VM formation and the β-catenin signaling pathway. Moroidin significantly reduced the level of p-ERK in U87 and U251 cells but did not affect the total expression level of ERK (***Fig. 5A***–***5D***), indicating that moroidin inhibited the activation of ERK. Studies have reported that the ERK inhibitor decreased both active β-catenin and MMP-9, the downstream molecule of EMT, which indicated the involvement of ERK in the EMT signaling pathway^[19]^.

### β-catenin agonist reversed the inhibitory effect of moroidin on VM

**Figure 6:**
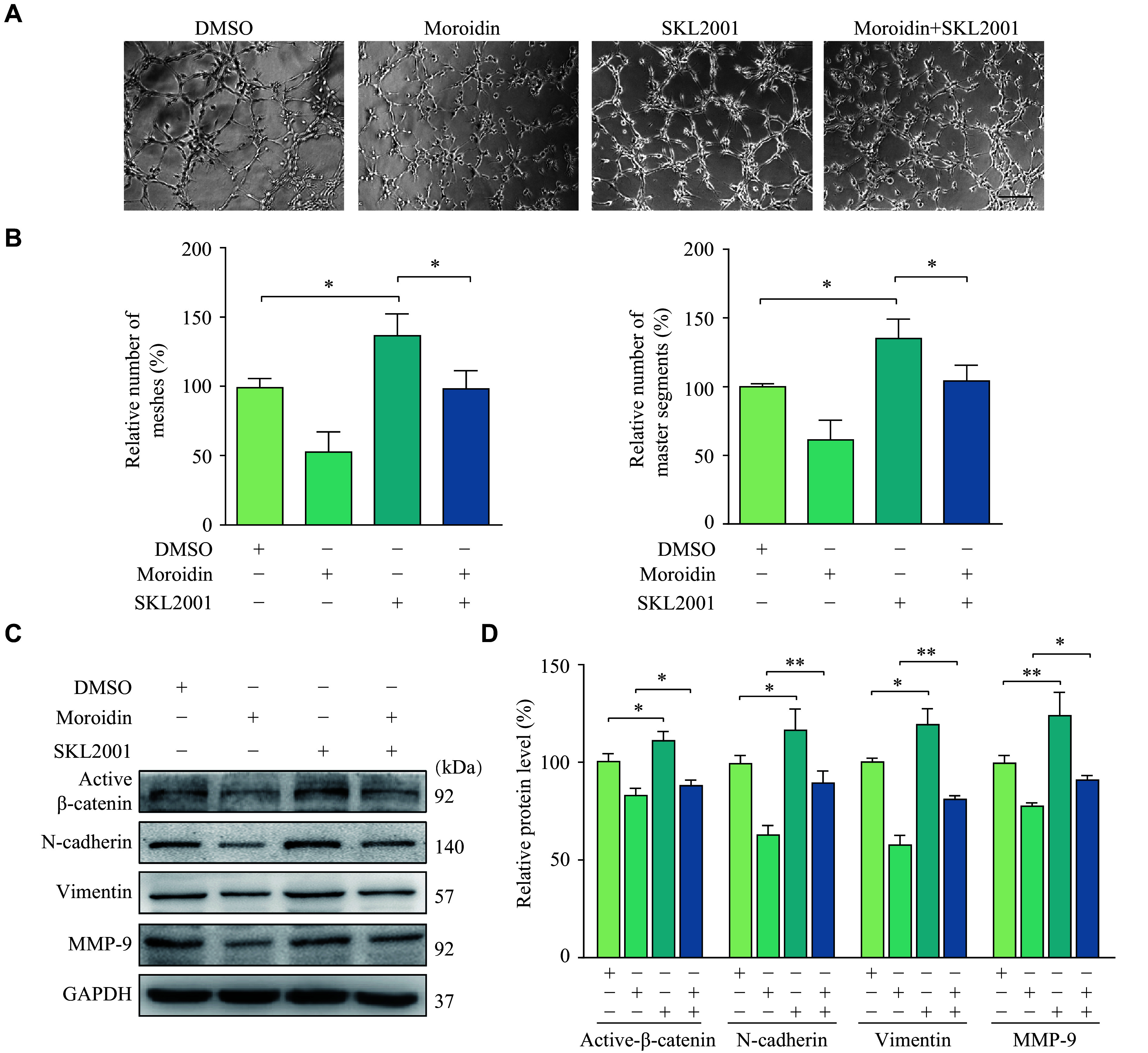
The activation of β-catenin abolished the effects of moroidin on glioblastoma cells.

**Figure 7:**
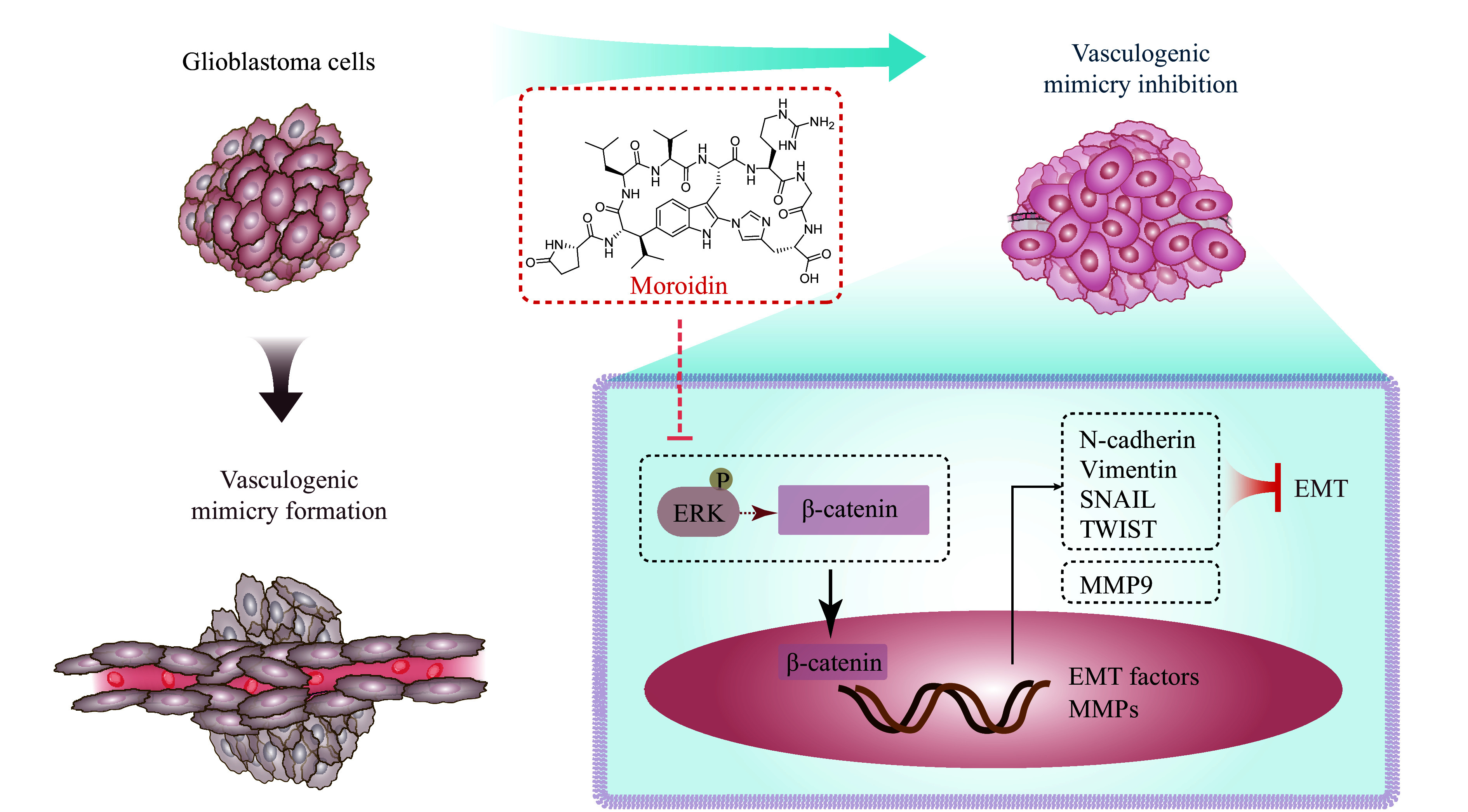
Pharmacological mechanisms of moroidin against vasculogenic mimicry formation of glioblastoma cells.

We used SKL2001, a β-catenin agonist, to further validate the role of the β-catenin/EMT pathway in the moroidin-mediated VM disruption. As shown in ***Fig. 6A*** and ***6B***, the inhibited tube formation induced by moroidin was significantly reversed in the presence of SKL2001, as evidenced by an increase in mesh and master segment numbers. More importantly, the moroidin-induced decrease in active β-catenin, N-cadherin, vimentin, and MMP-9 was rescued by SKL2001 (***Fig. 6C*** and ***6D***). Therefore, by inhibiting the activation of β-catenin, moroidin suppressed the EMT process in GBM cells and finally impaired VM formation (***Fig. 7***).

## Discussion

VM has been proven to be the most important supplementary form of tumor vascularization, especially under hypoxia and nutrient deficiency induced by AAT^[20–22]^. Targeting VM represents a promising strategy to overcome AAT resistance and control GBM recurrence. In the present study, we found that cyclopeptide moroidin significantly inhibited VM formation in GBM cells *in vitro* at non-cytotoxic concentrations. Furthermore, the inhibition of β-catenin and EMT pathway was a mechanism underlying the anti-VM effect of moroidin.

Some accumulating evidence suggests that plant-derived natural products target VM structures by influencing multiple signaling pathways. The search for high-efficacy and low-toxicity prodrugs with anti-VM activity from plant-derived products has attracted much attention. The unique cyclic structure imparts structural rigidity and biological stability to the plant cyclopeptides, making them an ideal molecular scaffold for the development of novel antitumor drugs^[23]^. Despite its satisfactory activity and novel structure, the natural cyclopeptide moroidin has barely been investigated. Here, we report for the first time that moroidin inhibits *in vitro* migration, invasion, and VM formation of GBM cells at sublethal concentrations, a new function that differs from the previous studies^[16]^. Inconsistent with the previous reports^[24]^, we found that BEV enhanced VM formation, which was disrupted by moroidin. These results indicate the potential of moroidin as a useful supplement for the anti-angiogenic therapy of GBM.

To further study the action pattern, we performed transcriptomic analysis and found that, in addition to genes involved in microtubule polymerization, genes involved in the biological processes of epithelial cell development, cell morphogenesis, and cell-cell adhesion were significantly enriched by the GO analysis, which suggests a new function of moroidin. Furthermore, the GSEA data showed that the EMT signaling pathway, especially the tumor invasion-associated genes, was significantly downregulated after treatment with moroidin. EMT is a dynamically reversible biological process involving the transdifferentiation of epithelial cells into quasi-mesenchymal cells, which are essential for tumor invasion, VM formation, and drug resistance. After treatment with moroidin, the levels of mesenchymal state markers, including N-cadherin, vimentin, SNAIL, and TWIST, decreased in a dose-dependent manner, while E-cadherin expression level was increased, demonstrating that moroidin inhibited EMT in GBM cells. Moroidin significantly inhibited cell motility and Matrigel tubule formation, altered cell morphology, and downregulated the expression of MMP-9 in GBM cells, thereby disrupting VM formation.

We further detected the activation of β-catenin, a key upstream transcriptional regulator that triggers the expression of TWIST and SNAIL. Moroidin inhibited β-catenin activation and nuclear translocation without suppressing the total expression level of β-catenin. Therefore, these results demonstrate that moroidin suppresses the VM formation by reducing the β-catenin-mediated EMT process in GBM cells.

In contrast, ERK was found to regulate both EMT and VM in GBM cells^[25]^. Moreover, the ERK inhibitor, U0126, significantly inhibited ERK phosphorylation and decreased the expression levels of active β-catenin and MMP-9^[19]^. Therefore, ERK may be involved in the inhibitory effects of moroidin on β-catenin activation and EMT in GBM cells.

Previous studies identified moroidin as a microtubule targeting agent and calculated the inhibitory IC_50_ value of moroidin on the tubulin polymerization to be 3.0 μmol/L^[14–15]^. The present data from the transcriptomic analysis also indicated the involvement of microtubule association in the moroidin-induced biological events. However, we focused on the novel effect of moroidin on the VM formation in GBM cells at lower concentrations, which is distinct from the previous reports. Our previous study indicated that moroidin also induced cytotoxicity in U87 and U251 cells when incubated for a longer period, with IC_50_ values of 9.6 and 5.2 μmol/L, respectively^[16]^. We speculate that moroidin may play a far more powerful and complex anti-tumor role when used *in vivo* by simultaneously inhibiting angiogenesis and directly killing cells.

Although these results were encouraging, it was very frustrating that the *in vivo* evaluation could hardly proceed because of the low isolation yields of moroidin from source plants. Another controversial question was whether moroidin could overcome the blood-brain barrier. Moroidin may reach GBM tumors under the presence of a blood-tumor barrier that is more permeable than the blood-brain barrier. On the other hand, cyclized structures may improve biological membrane permeability and passive diffusion^[26]^. There is still a need for much more solid evidence to further elucidate the characteristics of moroidin.

In conclusion, for the first time, we demonstrated the therapeutic effect of moroidin by inhibiting VM in GBM cells. Moroidin suppressed the motility, migration, and subsequent VM formation at sub-lethal concentrations by regulating the ERK/β-catenin-dependent EMT pathway. The present study reveals a new functional pattern and mechanism of this herbal cyclopeptide and provides a potential candidate for anti-angiogenic therapy for GBM.

## SUPPLEMENTARY DATA

Supplementary data to this article can be found online.

## References

[1] (2020). Management of glioblastoma: State of the art and future directions. CA Cancer J Clin.

[2] (2021). Glioblastoma multiforme (GBM): An overview of current therapies and mechanisms of resistance. Pharmacol Res.

[3] (2020). The role of selected chemokines and their receptors in the development of gliomas. Int J Mol Sci.

[4] (2018). Anti-angiogenic therapy for high-grade glioma. Cochrane Database Syst Rev.

[5] (2017). Vasculogenic mimicry signaling revisited: focus on non-vascular VE-cadherin. Mol Cancer.

[6] (2017). Structural and functional identification of vasculogenic mimicry *in vitro*. Sci Rep.

[7] (2023). Flavokawain A suppresses the vasculogenic mimicry of HCC by inhibiting CXCL12 mediated EMT. Phytomedicine.

[8] (2005). Does vasculogenic mimicry exist in astrocytoma?. J Histochem Cytochem.

[9] (2020). Association between glioblastoma cell-derived vessels and poor prognosis of the patients. Cancer Commun.

[10] (2021). Vasculogenic mimicry, a complex and devious process favoring tumorigenesis—Interest in making it a therapeutic target. Pharmacol Ther.

[11] (2022). Prrx1 promotes resistance to temozolomide by upregulating ABCC1 and inducing vasculogenic mimicry in glioma. Am J Cancer Res.

[12] (2021). Regulatory mechanisms and therapeutic targeting of vasculogenic mimicry in hepatocellular carcinoma. Pharmacol Res.

[13] (2021). The role of daurisoline treatment in hepatocellular carcinoma: Inhibiting vasculogenic mimicry formation and enhancing sensitivity to sorafenib. Phytomedicine.

[14] (2001). Celogentins A-C, new antimitotic bicyclic peptides from the seeds of *Celosia argentea*. J Org Chem.

[15] (2000). Antimitotic activity of moroidin, a bicyclic peptide from the seeds of *Celosia argentea*. Bioorg Med Chem Lett.

[16] (2022). Moroidin, a cyclopeptide from the seeds of *Celosia cristata* that induces apoptosis in A549 human lung cancer cells. J Nat Prod.

[17] (2024). Drug resistance mechanisms in cancers: Execution of pro-survival strategies. J Biomed Res.

[18] (2019). MICAL-L2 potentiates Cdc42-dependent EGFR stability and promotes gastric cancer cell migration. J Cell Mol Med.

[19] (2019). Blockade of ERK1/2 by U0126 alleviates uric acid-induced EMT and tubular cell injury in rats with hyperuricemic nephropathy. Am J Physiol Renal Physiol.

[20] (2020). The role of microenvironment in tumor angiogenesis. J Exp Clin Cancer Res.

[21] (2020). Targeting vasculogenic mimicry by phytochemicals: a potential opportunity for cancer therapy. IUBMB Life.

[22] (2021). Glioma stem cells and their roles within the hypoxic tumor microenvironment. Theranostics.

[23] (2022). Cyclic peptide drugs approved in the last two decades (2001–2021). RSC Chem Biol.

[24] (2017). Autophagy-induced KDR/VEGFR-2 activation promotes the formation of vasculogenic mimicry by glioma stem cells. Autophagy.

[25] (2021). Tenascin-c knockdown suppresses vasculogenic mimicry of gastric cancer by inhibiting ERK-triggered EMT. Cell Death Dis.

[26] (2019). Understanding cell penetration of cyclic peptides. Chem Rev.

