# Supplementary material for: Cyclopeptide moroidin inhibits vasculogenic mimicry formed by glioblastoma cells via regulating β-catenin activation and EMT pathways
Source: J Biomed Res. 2024 May 29;38(4):322–33. doi: 10.7555/JBR.38.20240015 (PMC11300521; doi:10.7555/JBR.38.20240015)
Supplement: Supplementary file 1 — Supplementary data to this article can be found online. [file jbr-38-4-322-S1.pdf]

Available online at www.jbr-pub.org.cn

Open Access at PubMed Central

JBR

Journal of Biomedical Research, 2024 38(4): 1–2

Supplementary Data

Cyclopeptide moroidin inhibits vasculogenic mimicry formed by glioblastoma cells *via* regulating β-catenin activation and EMT pathways

Pengxiang Min[1,△], Yingying Li[1,△], Cuirong Wang[1,△], Junting Fan[2,△], Shangming Liu[1], Xiang Chen[1], Yamin Tang[3], Feng Han[1,4,✉], Aixia Zhang[5,✉], Lili Feng[1,✉]

[1]*Key Laboratory of Cardiovascular & Cerebrovascular Medicine, International Joint Laboratory for Drug Target of Critical Illnesses, School of Pharmacy, Nanjing Medical University, Nanjing, Jiangsu 211166, China;*
[2]*Department of Pharmaceutical Analysis, School of Pharmacy, Nanjing Medical University, Nanjing, Jiangsu 210029, China;*
[3]*Department of Analysis and Testing Center, School of Basic Medical Sciences, Nanjing Medical University, Nanjing, Jiangsu 211166, China;*
[4]*Institute of Brain Science, the Affiliated Brain Hospital of Nanjing Medical University, Nanjing, Jiangsu 211166, China;*
[5]*Department of Clinical Pharmacology, School of Pharmacy, Nanjing Medical University, Nanjing, Jiangsu 211166, China.*

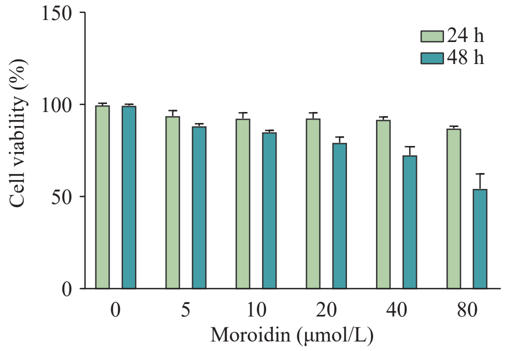

Supplementary Fig. 1 **Effect of moroidin on human umbilical vein endothelial cells (HUVECs).** HUVECs were treated with moroidin (0, 5, 10, 20, 40, and 80 μmol/L) for 24 h and 48 h, respectively. Cell viability was analyzed by CCK-8 assay.

△These authors contributed equally to this work.

✉Corresponding authors: Feng Han, Key Laboratory of Cardiovascular & Cerebrovascular Medicine, Institute of Brain Science, the Affiliated Brain Hospital of Nanjing Medical University, School of Pharmacy, Nanjing Medical University, 101 Longmian Avenue, Jiangning District, Nanjing, Jiangsu 211166, China. E-mail: fenghan169@njmu.edu.cn; Aixia Zhang, Department of Clinical Pharmacology, School of Pharmacy, Nanjing Medical University, 101 Longmian Avenue, Jiangning District, Nanjing, Jiangsu 211166, China. E-mail: aixia.zhang@njmu.edu.cn; Lili Feng, Key Laboratory of Cardiovascular & Cerebrovascular Medicine, International Joint Laboratory for Drug Target of Critical Illnesses, School of Pharmacy, Nanjing Medical University, 101 Longmian Avenue, Jiangning District, Nanjing, Jiangsu 211166, China. E-mail: fenglilinjmu@njmu.edu.cn.

Received: 19 January 2024; Revised: 30 April 2024; Accepted: 10 May 2024; Published online: 29 May 2024

CLC number: R739.41, Document code: A

The authors reported no conflict of interests.

This is an open access article under the Creative Commons Attribution (CC BY 4.0) license, which permits others to distribute, remix, adapt and build upon this work, for commercial use, provided the original work is properly cited.

© 2024 by Journal of Biomedical Research.

https://doi.org/10.7555/JBR.38.20240015

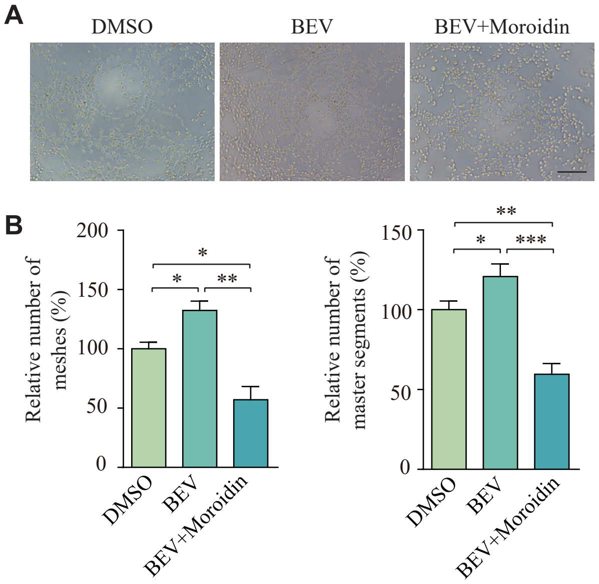

Supplementary Fig. 2 **Effect of moroidin on bevacizumab (BEV) enhanced vasculogenic mimicry formation.** A: U87 cells were treated with BEV or BEV + moroidin and then seeded onto the surface of matrigel. Structures formed by cells were imaged using a microscope. Scale bar, 200 μm. B: Statistical analysis of the relative number of meshes and master segments. $^{*}P < 0.05$, $^{**}P < 0.01$, and $^{***}P < 0.001$ compared with the DMSO group by one-way ANOVA followed by Dunnett's post hoc test. Abbreviation: DMSO, dimethyl sulfoxide.

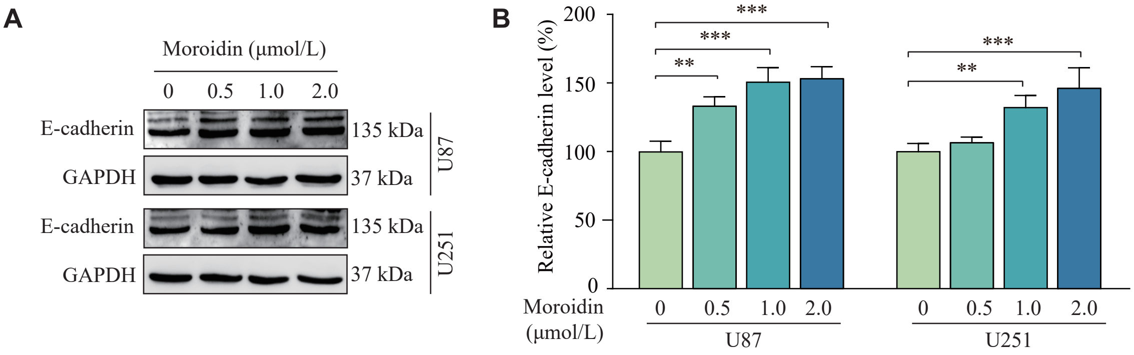

Supplementary Fig. 3 **Moroidin up-regulated the expression of E-cadherin.** A: U87 and U251 cells were treated with moroidin for 24 h. Protein levels of E-cadherin were determined by Western blotting. B: Quantitative analysis of protein levels. $^{*}P < 0.05$ compared with the control group by two-way ANOVA followed by Dunnett's post hoc test.

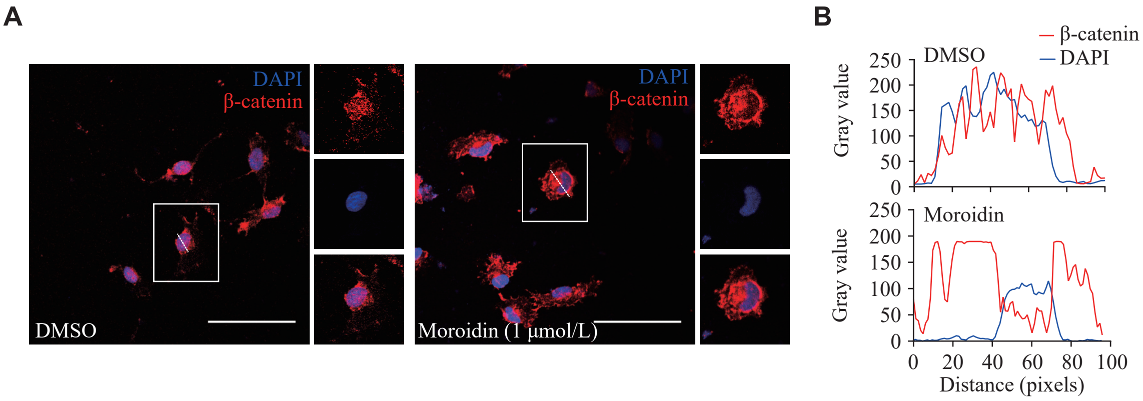

Supplementary Fig. 4 **Moroidin repressed the translocation of β-catenin in the nucleus.** A: U251 cells were treated with moroidin for 24 h. The nuclear translocation of β-catenin was detected using the immunofluorescence assay. Scale bar, 100 μm. B: The line charts represent the gray value of the β-catenin and DAPI.
